# The Phylogenomic Diversity of Herbivore-Associated *Fibrobacter* spp. Is Correlated to Lignocellulose-Degrading Potential

**DOI:** 10.1128/mSphere.00593-18

**Published:** 2018-12-12

**Authors:** Anthony P. Neumann, Garret Suen

**Affiliations:** a Department of Bacteriology, University of Wisconsin—Madison, Madison, Wisconsin, USA; University of California, Davis

**Keywords:** *Fibrobacter*, carbohydrate-active enzymes, cellulose, fiber, genomics, gut microbiota, herbivores

## Abstract

Members of the genus *Fibrobacter* are cellulose-degrading bacteria and common constituents of the gastrointestinal microbiota of herbivores. Although considerable phylogenetic diversity is observed among members of this group, few functional differences explaining the distinct ecological distributions of specific phylotypes have been described. In this study, we sequenced and performed a comparative analysis of whole genomes from 38 novel *Fibrobacter* strains against the type strains for the two formally described *Fibrobacter* species F. succinogenes strain S85 and F. intestinalis strain NR9. Significant differences in the number of genes encoding carbohydrate-active enzyme families involved in plant cell wall polysaccharide degradation were observed among *Fibrobacter* phylotypes. F. succinogenes genomes were consistently enriched in genes encoding carbohydrate-active enzymes compared to those of F. intestinalis strains. Moreover, genomes of F. succinogenes phylotypes that are dominant in the rumen had significantly more genes annotated to major families involved in hemicellulose degradation (e.g., CE6, GH10, and GH43) than did the genomes of F. succinogenes phylotypes typically observed in the lower gut of large hindgut-fermenting herbivores such as horses. Genes encoding a putative urease were also identified in 12 of the *Fibrobacter* genomes, which were primarily isolated from hindgut-fermenting hosts. Screening for growth on urea as the sole source of nitrogen provided strong evidence that the urease was active in these strains. These results represent the strongest evidence reported to date for specific functional differences contributing to the ecology of *Fibrobacter* spp. in the herbivore gut.

**IMPORTANCE** The herbivore gut microbiome is incredibly diverse, and a functional understanding of this diversity is needed to more reliably manipulate this community for specific gain, such as increased production in ruminant livestock. Microbial degraders of plant cell wall polysaccharides in the herbivore gut, particularly *Fibrobacter* spp., are of fundamental importance to their hosts for digestion of a diet consisting primarily of recalcitrant plant fibers. Considerable phylogenetic diversity exists among members of the genus *Fibrobacter*, but much of this diversity remains cryptic. Here, we used comparative genomics, applied to a diverse collection of recently isolated *Fibrobacter* strains, to identify a robust association between carbohydrate-active enzyme gene content and the *Fibrobacter* phylogeny. Our results provide the strongest evidence reported to date for functional differences among *Fibrobacter* phylotypes associated with either the rumen or the hindgut and emphasize the general significance of carbohydrate-active enzymes in the evolution of fiber-degrading bacteria.

## Abstract (precis)

The herbivore gut microbiome is incredibly diverse, and a functional understanding of this diversity is needed to more reliably manipulate this community for specific gain, such as increased production in ruminant livestock. Microbial degraders of plant cell wall polysaccharides in the herbivore gut, particularly *Fibrobacter* spp., are of fundamental importance to their hosts for digestion of a diet consisting primarily of recalcitrant plant fibers. Considerable phylogenetic diversity exists among members of the genus *Fibrobacter*, but much of this diversity remains cryptic. Here, we used comparative genomics, applied to a diverse collection of recently isolated *Fibrobacter* strains, to identify a robust association between carbohydrate-active enzyme gene content and the *Fibrobacter* phylogeny. Our results provide the strongest evidence reported to date for functional differences among *Fibrobacter* phylotypes associated with either the rumen or the hindgut and emphasize the general significance of carbohydrate-active enzymes in the evolution of fiber-degrading bacteria.

## INTRODUCTION

Herbivorous animals are capable of living on a diet of recalcitrant plant cell wall polysaccharides (PCWP) because of a symbiotic association with fibrolytic microbial communities residing in their gastrointestinal tract (1). Herbivorous vertebrates generally lack the ability to hydrolyze PCWP themselves and are therefore entirely dependent on their gut microbes to degrade and ferment these substrates into short-chain fatty acids, which they can then use for energy (2). Efficient digestion of PCWP requires a complex and dynamic microbiota because the plant cell wall has evolved a structure that is heterogeneous, chemically diverse, and highly resistant to depolymerization (3). The plant cell wall consists of cellulose fibers embedded in a matrix of hemicellulose, pectin, and lignin, and fibrolytic microbes produce a suite of enzymes that work synergistically to degrade these different classes of organic polymers (4). These carbohydrate-active enzymes (CAZymes) have been classified into numerous classes and families based on their structure and homology (5, 6).

The major classes of catalytic CAZymes include the following: glycoside hydrolases (GHs), glycosyltransferases (GTs), polysaccharide lyases (PLs), carbohydrate esterases (CEs), and auxiliary activities (AAs) (7). GHs hydrolyze glycosidic bonds in polysaccharides, in either an “endo” or “exo” manner. GTs catalyze the formation of glycosidic linkages from activated carbohydrate precursors. PLs perform nonhydrolytic cleavage of polysaccharides through a β-elimination-type reaction mechanism. CEs hydrolyze carbohydrate esters and include enzymes for liberating acetyl and phenolic esters which are common constituents of hemicelluloses. AAs encompass enzymes involved in redox reactions that enhance the activity of other catalytic CAZymes. Catalytic CAZyme modules are often covalently linked to a noncatalytic module that has an affinity for binding certain carbohydrate moieties. These carbohydrate-binding modules (CBMs) enhance the efficiency of their catalytic partners by ensuring close contact between the enzyme and its target substrate. Multiple enzyme families from most, if not all, of these CAZyme classes are produced by the most actively fibrolytic microorganisms in plant-digesting microbial communities such as the rumen (8 – 11).

Bacteria possessing a superior ability to solubilize PCWP are often particularly well suited to utilization of cellulose for growth (12, 13). Cellulose is commonly the most abundant structural polysaccharide in plant matter, but it is also among the most resistant to degradation (14). The relationship between herbivores and cellulose-utilizing bacteria is best understood in the context of the bovine rumen, where species of *Ruminococcus* and *Fibrobacter* are the primary degraders (15). *Fibrobacter* is a conserved member of the rumen microbiota, and *Fibrobacter* species possess several characteristics that contribute to efficient rumen function (16). Although *Fibrobacter* species readily solubilize hemicelluloses and pectins, available evidence indicates that energy generation in these bacteria results exclusively from the catabolism of hexoses and that these bacteria preferentially target cellulose as a substrate for growth (17 – 19). As a result, xylooligosaccharides and pentoses liberated from insoluble forms of hemicellulose and pectin, respectively, via *Fibrobacter* become available for consumption by other rumen microbes. *Fibrobacter* also contributes to rumen efficiency through the production of succinate, the primary fermentation product produced by these bacteria (20). Succinate does not accumulate in the rumen and is converted primarily to propionate, an important substrate used by the host for gluconeogenesis (21 – 23). This pathway preserves reducing equivalents for energy generation by the host that might otherwise be lost to methane production via the consumption of hydrogen, an alternative fermentation product, by methanogens (24).

Since being identified in the rumen, evidence for diverse populations of *Fibrobacter* and related bacteria in the phylum *Fibrobacteres* has accumulated as a result of culture-independent studies (25 – 28). However, evidence of functional differences explaining much of this diversity is lacking, due, at least in part, to a general absence of axenic cultures available for study (29). Two species, F. intestinalis and F. succinogenes, have been formally described, but these species are not reliably differentiated using traditional characterization techniques and available representative strains (30). Comparative genomics of *Fibrobacteres* genomes recovered from metagenomic surveys has recently demonstrated the utility of this approach for gaining functional insights into phylogenetic and ecological differences among the members of this poorly understood phylum (31).

Recently, we reported a novel isolation technique that was used to greatly expand the diversity of *Fibrobacter* strains available for study as well as to provide additional evidence strengthening the association of certain phylotypes with the rumen or the hindgut (17). Here, we build upon our previous work by making functional predictions explaining the ecological basis of diversity among *Fibrobacter* spp. in the herbivore gut using whole-genome comparisons of our previously described *Fibrobacter* isolates. We hypothesized that adaptations to specific gastrointestinal compartments, as well as variations in PCWP-degrading potential, explain the differences in ecological distributions observed among *Fibrobacter* phylotypes. Our results indicate a strong association between the *Fibrobacter* phylogeny and CAZyme gene content, which likely contributes to the ecology of these bacteria, especially the dominance of certain phylotypes in the rumen.

## RESULTS

A total of 40 *Fibrobacter* genomes from axenic cultures were analyzed in this study, including type strains F. succinogenes S85 and F. intestinalis NR9 as well as 38 strains recently isolated and sequenced from diverse herbivores (see Table S1 in the supplemental material) (17). Overall, 16 of the *Fibrobacter* genomes were from strains isolated from rumen samples, while the remaining 24 were from strains isolated from the feces or cecal contents of hindgut-fermenting herbivores. The 40 strains were previously identified as phylogenetically related to F. succinogenes (*n* = 33) and F. intestinalis (*n* = 7) (17). Although a draft genome sequence for the F. intestinalis type strain, NR9, was publicly available prior to this work, the strain was resequenced with the aim of improving the assembly. This resulted in an improved draft of NR9, consisting of a single scaffold, which was subsequently utilized for all genomic analyses of NR9 in this study (Table S2). Overall, the genomes are estimated to be ≥98.8% complete, with all the assemblies consisting of 100 or fewer scaffolds (Table S2). The estimated sizes of the genomes range from 2.85 to 4.02 Mbp (mean, 3.56 ± 0.28 Mb). The GC content of the genomes ranges from 44.8% to 53.8% (mean, 49.3% ± 2.1%).

10.1128/mSphere.00593-18.1 TABLE S1 Fibrobacter strains used for comparative genomics. Download Table S1, PDF file, 0.1 MB. Copyright © 2018 Neumann and Suen. 2018 Neumann and Suen This content is distributed under the terms of the Creative Commons Attribution 4.0 International license.

10.1128/mSphere.00593-18.2 TABLE S2 Genome assembly statistics. Download Table S2, PDF file, 0.1 MB. Copyright © 2018 Neumann and Suen. 2018 Neumann and Suen This content is distributed under the terms of the Creative Commons Attribution 4.0 International license.

### *Fibrobacter* strains from herbivores are phylogenetically diverse.

To understand the phylogenetic diversity of our *Fibrobacter* genome collection, we conducted pairwise comparisons between all genomes using average nucleotide identity (ANI). The mean and median ANI values for all comparisons were 82.5% ± 4.7% and 80.7%, respectively. Strains UWR1 and UWR4 were the most closely related based on ANI (99.9%), while strains NR9 and UWH5 were the most divergent (76.1%). The 40 *Fibrobacter* strains were clustered based on ANI distance and were grouped using a cutoff of 95% ANI in order to estimate the total number of individual species-level groups present (32). Our results identified 21 different species-level groups among the 40 *Fibrobacter* strains using these criteria (Table 1). The largest of these groups contained 6 strains isolated from fecal samples originating from horses and a tapir. No other group contained more than 3 strains. Ten of the 21 groups were represented by only a single strain.

**TABLE 1:** *Fibrobacter* strain characteristics^*a*^

Strain	95% ANI group	d-Lactose	Urea
UWB1	3	ND	ND
UWB2	4	ND	ND
UWB3	5	+	ND
UWB4	6	+	ND
UWB5	7	ND	ND
UWB6	13	ND	ND
UWB7	12	+	+
UWB8	13	ND	ND
UWB10	21	ND	+
UWB11	11	+	ND
UWB12	11	ND	ND
UWB13	12	ND	ND
UWB15	13	ND	ND
UWB16	5	ND	ND
UWCM	14	ND	ND
UWEL	15	ND	+
UWH1	8	ND	+
UWH3	8	ND	+
UWH4	16	ND	ND
UWH5	8	ND	+
UWH6	8	ND	+
UWH8	8	ND	+
UWH9	8	+	+
UWOS	1	ND	ND
UWOV1	3	ND	ND
UWP2	17	ND	ND
UWR1	19	ND	+
UWR2	9	ND	ND
UWR3	14	ND	ND
UWR4	19	ND	ND
UWRM	20	ND	ND
UWS1	1	ND	ND
UWS2	10	ND	ND
UWS3	10	ND	ND
UWS4	20	ND	ND
UWT1	8	ND	+
UWT2	18	ND	+
UWT3	14	ND	ND
S85	2	+	ND
NR9	1	ND	ND

a All strains grew on d-glucose and d-cellobiose. No strains grew on l-arabinose, d-mannose, d-xylose, d-galactose, d-maltose, or l-rhamnose. ANI, average nucleotide identity; +, growth observed; ND, growth not detected.

**FIG 1:**
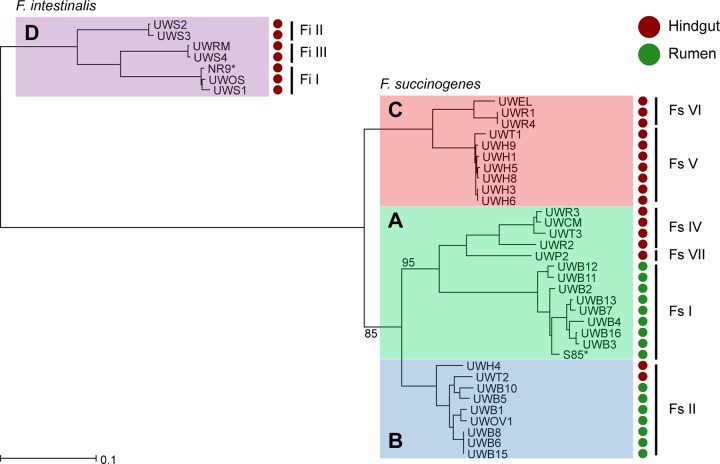
A multilocus phylogeny of *Fibrobacter* strains. The maximum likelihood phylogeny was constructed from the concatenated alignments of 99 essential protein genes using RAxML (68) and is midpoint rooted. Strains are annotated with circles according to their isolation source, rumen (dark green) or hindgut (dark red). Previously assigned *Fibrobacter* phylotypes are labeled, and their coverage of the tree is marked with black vertical bars (17). Type strains are identified with an asterisk (*). Major clades are shaded according to the following clade designations: clade A, green; clade B, blue; clade C, red; clade D, violet. The bootstrap value for clades containing more than 5 strains was 100%, unless otherwise indicated (100 replicates). The scale represents the number of substitutions per site.

To better resolve the phylogeny of our strains, we constructed a multilocus sequence tree from concatenated alignments of protein sequences encoded by single-copy essential genes (33) (Fig. 1). The 40 *Fibrobacter* strains clearly segregated into two groups with one group containing the 7 F. intestinalis strains and the other containing the 33 F. succinogenes strains. The strains were further resolved into 4 readily distinguishable major clades, 3 of which contained all strains of F. succinogenes, with the remaining clade containing all 7 strains of F. intestinalis. The 4 major clades were designated *Fibrobacter* clades A to D (Fig. 1). *Fibrobacter* clade A contained strains isolated from the rumen, as well as from hindgut fermenters, and consisted of previously described phylotypes Fs I, Fs IV, and Fs VII (17). Clade B also contained rumen and hindgut isolates and contained members of the previously described phylotype Fs II. Clade C contained strains of phylotypes Fs V and Fs VI, found exclusively in the hindgut. Clade D consisted of all the F. intestinalis strains examined in this study, which were further resolved into three discrete groups corresponding to the previous designations of Fi I, Fi II, and Fi III.

### A conserved general mechanism for PCWP degradation by *Fibrobacter* spp.

To better understand the lignocellulose-degrading potential of these *Fibrobacter* strains, we annotated our genomes using the carbohydrate-active enzyme (CAZyme) database. The total number of CAZymes predicted in the genomes ranged from 120 for UWOS to 219 for UWT2. The average number of total CAZymes predicted in a single *Fibrobacter* genome was 190 ± 30.4, and the median was 198. Mean gene counts per genome for CAZyme classes predicted to play a role in PCWP degradation by *Fibrobacter* spp. were as follows: 85.1 ± 17.3 for glycoside hydrolases (GHs), 35.0 ± 7.6 for carbohydrate-binding modules (CBMs), 15.2 ± 4.1 for carbohydrate esterases (CEs), and 12.5 ± 2.6 for polysaccharide lyases (PL). No genes predicted to code for CAZyme auxiliary activities were identified in any of the *Fibrobacter* genomes.

**FIG 2:**
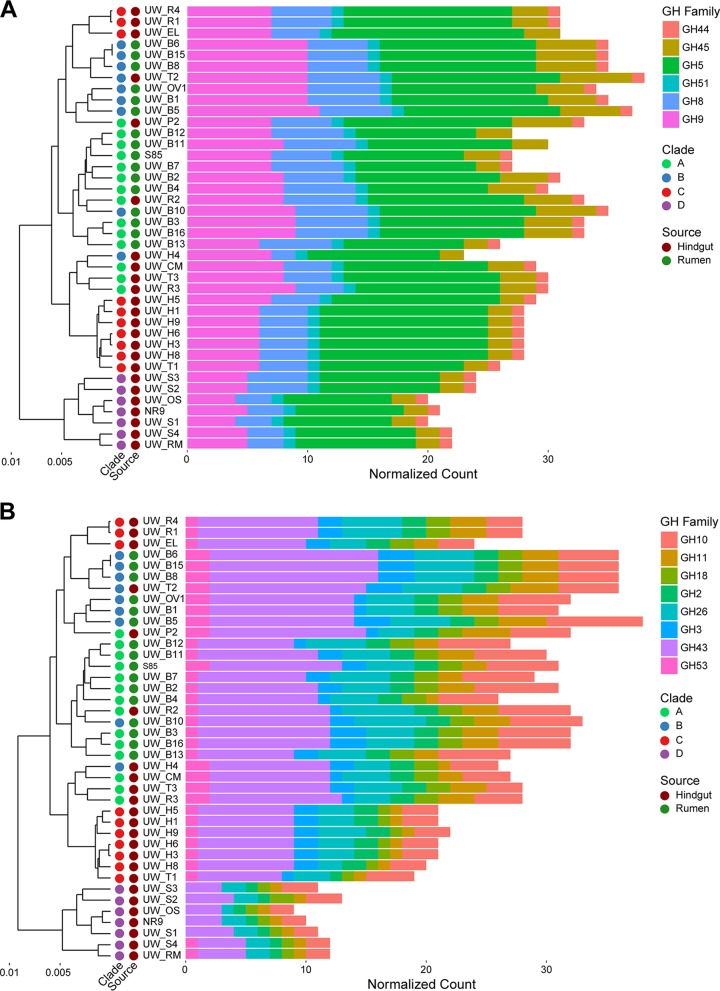
Distribution of cellulase (A) and hemicellulase (B) CAZyme families among *Fibrobacter* strains. *Fibrobacter* strains are arranged according to the results of hierarchical clustering by relative abundance of all CAZyme family genes in their respective genomes. *Fibrobacter* strains are annotated with circles according to phylogenetic clade membership as clade A (green), clade B (blue), clade C (red), and clade D (violet) and according to isolation source as rumen (dark green) or hindgut (dark red). Horizontal bar plots extend along the *x* axis according to the number of normalized gene counts in each glycoside hydrolase (GH) family. GH families are color coded separately for cellulases (A) and hemicellulases (B).

An analysis of the specific CAZyme families identified in the genomes indicated that they were generally consistent among strains. Of the 72 CAZyme families identified across all genomes, 39 (54.2%) were present in all *Fibrobacter* strains (Table S3). These 39 conserved CAZyme families accounted for 87.6% of the total CAZyme genes identified. The GH families containing the major cellulases produced by *Fibrobacter* (18), GH5, GH8, GH9, and GH45, were relatively abundant and conserved in all *Fibrobacter* genomes (Fig. 2A). A single copy of the gene predicted to encode the major cellulose-binding endoglucanase, containing both a CBM11 and a GH51 module, was also identified in all 40 *Fibrobacter* genomes (34, 35). Similar trends were found for CAZymes implicated in hemicellulose degradation (Fig. 2B).

10.1128/mSphere.00593-18.3 TABLE S3 Summary stats for CAZyme families. Download Table S3, PDF file, 0.1 MB. Copyright © 2018 Neumann and Suen. 2018 Neumann and Suen This content is distributed under the terms of the Creative Commons Attribution 4.0 International license.

We found that CBM family 6 and CBM family 35 were the most abundant carbohydrate-binding modules present, regardless of strain, and were typically associated with hemicellulose-degrading CAZyme families, including CE6, GH10, and GH43. Additional proteins thought to assist in cellulose degradation by facilitating cellular adherence, i.e., proteins containing fibro-slime domains (34), were identified in all 40 genomes. The lowest number of fibro-slime domain-containing proteins predicted in a single genome was 3, which was observed for several strains of F. intestinalis. Most strains of F. succinogenes had 8 to 10 distinct fibro-slime domain-containing proteins. Finally, a single copy of the gene coding for a cellobiose/cellodextrin phosphorylase, an important enzyme for generating glucose-1-phosphate from cellodextrins liberated from the breakdown of cellulose, was also identified in all genomes.

### CAZyme genes are more abundant in F. succinogenes genomes.

**FIG 3:**
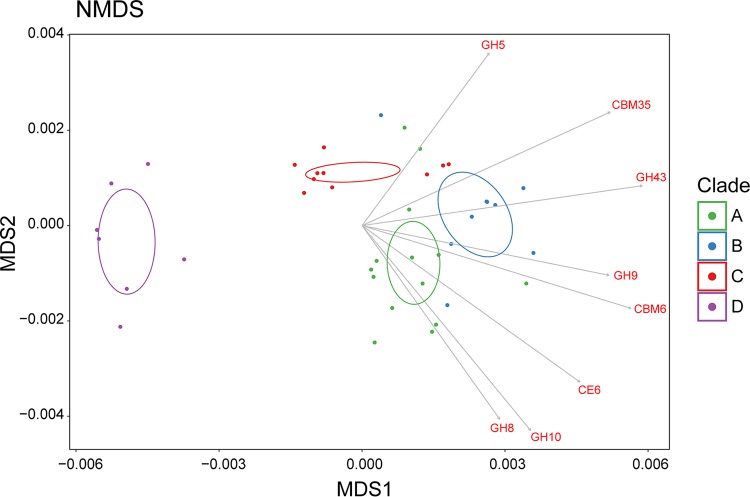
Scatterplot of 2-dimensional NMDS ordination based on the relative abundances of the genes for all CAZyme families in the *Fibrobacter* genomes. Individual points represent a single *Fibrobacter* strain, colored according to the phylogenetic clade, clade A (green), clade B (blue), clade C (red), and clade D (violet). Open standard error ellipses (95% confidence interval) are plotted for each *Fibrobacter* clade and are colored accordingly. Vectors for CAZyme families were calculated by fitting the relative abundances of those CAZyme families in the *Fibrobacter* genomes to the dimensions of the NMDS, with the largest significant differences among the clades plotted with gray arrows and labeled accordingly in red.

**FIG 4:**
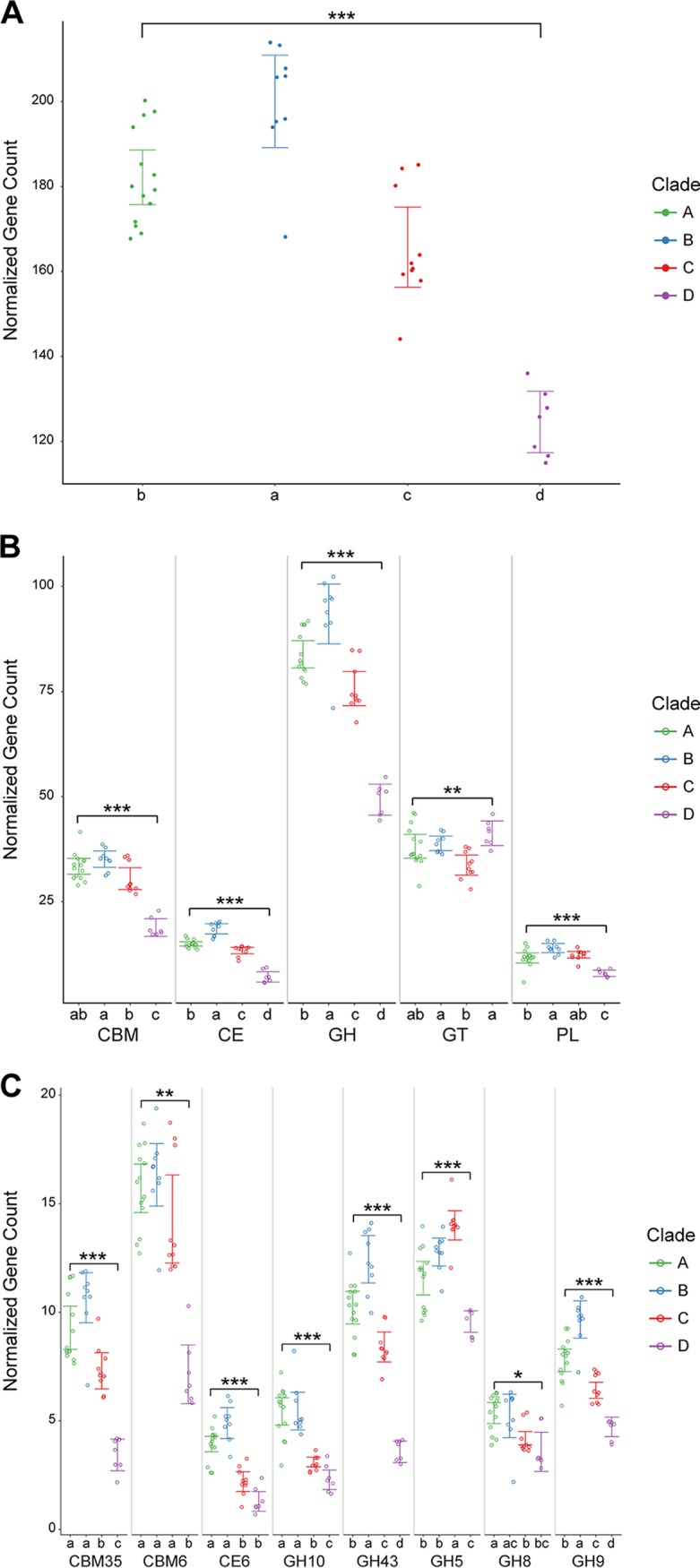
Significant differences in CAZyme gene counts among *Fibrobacter* clades. In each plot, a single point represents a single *Fibrobacter* strain, colored according to phylogenetic clade, clade A (green), clade B (blue), clade C (red), and clade D (violet). Error bars, colored according to clade, represent the 95% confidence interval for the mean normalized gene count for the clade. (A) Strip plot of normalized gene counts for total CAZymes in *Fibrobacter* genomes by phylogenetic clade. The bracket at the top of the plot represents the results of statistical testing for an overall difference among the clades with significance indicated as follows: •, *P < *0.05; *, *P < *0.01; **, *P < *0.001; ***, *P < *0.0001 (ANOVA). Lowercase letters along the bottom of each plot represent significantly different groups based on pairwise statistical tests using comparisons between individual clades (*t* test, adjusted *P* value [*P*_adj_] < 0.05 [Bonferroni’s correction]). (B) Strip plots of normalized gene counts for CAZyme classes. The plots are arranged horizontally by CAZyme class and labeled accordingly. Brackets represent the results of statistical testing for an overall difference among the clades for each CAZyme class, with significance indicated as follows: *P*_adj_ < 0.05 (·), *P*_adj_ < 0.01 (*), *P*_adj_ < 0.001 (**), *P*_adj_ < 0.0001 (***) (ANOVA, Bonferroni’s correction). Lowercase letters along the bottom of each plot represent significantly different groups based on pairwise statistical tests using comparisons between individual clades (*t* test, *P*_adj_ < 0.05 [Bonferroni’s correction]). (C) Strip plots of normalized gene counts for highly variable CAZyme families. The plots are arranged horizontally by CAZyme family and labeled accordingly. Brackets represent the results of statistical testing for an overall difference among the clades for each CAZyme family with significance indicated as follows: •, FDR < 0.05; *, FDR < 0.01; **, FDR < 0.001; ***, FDR < 0.0001 (Kruskal-Wallis rank sum test [71]). Lowercase letters along the bottom of each plot represent significantly different groups based on pairwise statistical tests using comparisons between individual clades (Wilcoxon rank sum test, *P*_adj_ < 0.05 [Bonferroni’s correction]).

An unsupervised statistical analysis of relationships among the 40 *Fibrobacter* strains based solely on the relative abundances of the CAZyme families identified in their respective genomes was performed in order to identify any association between CAZyme content and the *Fibrobacter* phylogeny, as determined from the sequences of single-copy essential genes (Fig. 1). Nonmetric multidimensional scaling (NMDS) analysis identified clear differences between the F. intestinalis genomes (clade D) and the F. succinogenes genomes (clades A to C) (Fig. 3). This observation was further supported by complete-linkage hierarchical clustering of the genomes by CAZyme gene content (Fig. 2). Plots of total CAZyme gene counts and counts of genes annotated to each CAZyme class, normalized to genome size, identified clear differences among the four major clades of our *Fibrobacter* phylogeny (Fig. 4). The largest differences were observed between the F. succinogenes clades (clades A to C) and the F. intestinalis clade (clade D). Significantly higher normalized gene counts were observed for each of F. succinogenes clades A, B, and C than for clade D for total CAZymes, CBMs, CEs, GHs, and PLs (Fig. 4; see also Table S4). A total of 21 of 72 (29.2%) individual CAZyme families had significantly greater gene counts in each of clades A, B, and C than in clade D (FDR < 0.05) (see Data Set S1 in the supplemental material). These included 14 of the 29 (48.3%) total GH families identified among all 40 of the *Fibrobacter* genomes. CBM family 77 and GH family 13 were the only CAZyme families significantly more abundant in clade D genomes than in either the clade A or clade B genomes, although these two families did not differ between the clade D and clade C genomes.

10.1128/mSphere.00593-18.4 TABLE S4 Statistical differences in CAZyme classes. Download Table S4, PDF file, 0.1 MB. Copyright © 2018 Neumann and Suen. 2018 Neumann and Suen This content is distributed under the terms of the Creative Commons Attribution 4.0 International license.

10.1128/mSphere.00593-18.6 DATA SET S1 List of CAZymes by family. Download Data Set S1, XLSX file, 0.02 MB. Copyright © 2018 Neumann and Suen. 2018 Neumann and Suen This content is distributed under the terms of the Creative Commons Attribution 4.0 International license.

### Enrichment in CAZyme genes in *Fibrobacter* clades containing rumen phylotypes.

Significantly greater total numbers of CAZyme genes, as well as of genes predicted to code for CEs and GHs, were observed in F. succinogenes clades A and B, which contain phylotypes that typically dominate in the rumen (17), than in clade C, which contains phylotypes that are rare in the rumen (Fig. 4; see also Table S4). *Fibrobacter* strains representative of rumen phylotypes generally had higher overall numbers of predicted cellulase and hemicellulase genes in their genomes. Upon fitting vectors representing the relative abundances of individual CAZyme families across the genomes to the dimensions of the NMDS analysis, several were identified that exhibited a strong positive association with areas of the scatterplot occupied by *Fibrobacter* strains of clade A and B (Fig. 3). Significantly higher normalized gene counts for CE6, GH8, GH9, GH10, GH43, and GH45 were observed in clade A and clade B genomes than in the clade C genomes (Fig. 4C; see also Data Set S1). Moreover, CBM35, which is often encoded by *Fibrobacter* genes also containing a hemicellulase module (18), was significantly enriched in strains in clades A and B relative to those in clade C (Fig. 4C). Significant differences in total numbers of CAZyme genes, CEs, and PLs were also observed between clades A and B, with the genomes from clade B having higher normalized gene counts in these categories (Fig. 4B; see also Table S4).

### Differences in carbon and nitrogen metabolism between rumen and hindgut phylotypes.

All 40 of the *Fibrobacter* genomes analyzed contained genes for a complete glycolytic pathway through the formation of phosphoenolpyruvate (PEP); however, the gene for pyruvate kinase was identified in the genomes of only 11 strains. These 11 strains were all isolated from hindgut-fermenting hosts, had phylogenetic placements in either *Fibrobacter* clade C or clade D, and included *Fibrobacter* strain UWEL as well as all strains previously classified in phylotypes Fs V and Fi I (17). All *Fibrobacter* genomes contained a highly conserved gene predicted to code for a PEP carboxykinase, which would allow the formation of oxaloacetate and nucleoside triphosphate from PEP. Genes for pyruvate carboxylase, acetyl-coenzyme A (acetyl-CoA) synthetase, phosphate acetyltransferase, and acetate kinase were also identified in all strains, though the genes for formate C-acetyltransferase were missing from all 10 strains in clade C. The genomes suggest that, similarly to other obligate anaerobes, all of the *Fibrobacter* strains use an incomplete, reductive tricarboxylic acid (TCA) cycle, since the genes for α-ketoglutarate dehydrogenase and succinyl-CoA synthetase are absent. All 40 *Fibrobacter* strains contain a gene predicted to code for a succinyl-CoA:acetate CoA-transferase and would therefore be capable of generating the essential metabolite succinyl-CoA via this enzyme. No strains had genes for enzymes of the oxidative branch of the pentose phosphate pathway, but all had genes predicted to encode enzymes of the nonoxidative branch.

The 40 *Fibrobacter* strains were screened for growth on a number of substrates and all were found to be capable of growth on d-glucose and d-cellobiose, in addition to microcrystalline cellulose in media with essential growth factors and NH_4_Cl as the sole nitrogen source (lacking yeast extract or tryptone). None of the strains grew on l-arabinose, d-mannose, d-xylose, d-galactose, d-maltose, or l-rhamnose. Six strains, including F. succinogenes type strain S85, exhibited growth on d-lactose (Table 1). All 40 *Fibrobacter* genomes contained genes predicted to encode β-galactosidase, but no genetic basis for the differences in d-lactose utilization could be readily identified. None of the 40 *Fibrobacter* genomes had genes coding for a lactose permease, the key enzyme responsible for lactose transport into the cell.

**FIG 5:**
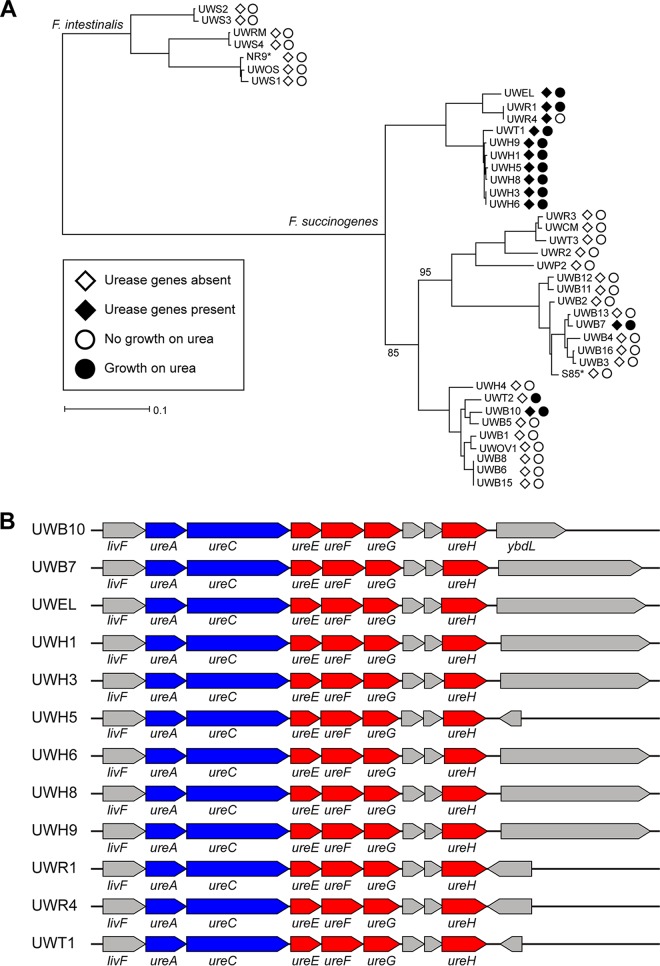
Presence of urease in *Fibrobacter* strains. (A) Maximum likelihood phylogeny constructed from the concatenated alignments of 99 essential protein genes with strains labeled according to the presence of urease genes and growth on urea as a source of nitrogen. Open diamonds (◊) represent the absence of the urease genes and filled diamonds (♦) represent the presence of urease genes in the genome. Open circles (○) indicate that the strain failed to grow in media with urea as the nitrogen source and filled circles (●) indicate that the strain was able to grow in media with urea as the source of nitrogen. (B) Aligned architecture of the approximately 5.7 kb region predicted to encode urease catalytic subunits (blue) and urease accessory proteins (red) in 12 positive strains. Genes not annotated as urease components are colored gray. Genes without labels are annotated as hypothetical proteins.

In addition to genes implicated in central carbon metabolism, the *Fibrobacter* genomes were analyzed for differences in genes predicted to be involved in nitrogen utilization. All 40 genomes had genes predicted to encode glutamate dehydrogenase and glutamine synthetase. We further identified an approximately 5.7-kbp region predicted to encode two urease catalytic subunits and 4 urease accessory proteins in 12 of the genomes, including all of the clade C genomes, and 2 phylogenetically distinct rumen strains, UWB7 and UWB10, from clades A and B, respectively (Fig. 5). Screening for growth on urea as the nitrogen source in media containing growth factors but lacking yeast extract or tryptone confirmed that 11 of these 12 strains were capable of using urea as the primary source of nitrogen (Table 1). Moreover, all other strains except one, UWT2, failed to grow in the media with urea as the sole nitrogen source, further supporting the idea of the presence of a functional urease in strains containing the predicted urease genes.

## DISCUSSION

Substantial phylogenetic diversity among bacteria in the genus *Fibrobacter* has been reported, but functional explanations for much of this diversity have proven elusive (26, 28, 29). We attempted to address this knowledge gap using whole-genome comparisons among the largest collection of *Fibrobacter* genomes from axenic cultures generated to date. Considerably more phylogenetic diversity was observed among the 40 *Fibrobacter* strains than previously estimated using full-length 16S rRNA gene sequences (17). Our finding of 21 different species-level groups, using whole-genome ANI (32), further resolves the 9 discrete phylotypes that we previously identified using the 16S rRNA gene alone (17). Our results are, however, consistent with what has generally been reported regarding differences in diversity estimates from whole-genome sequences compared to the 16S rRNA gene for other bacterial groups (36). It remains to be determined whether the 21 different species-level groups identified here are truly representative of ecologically discrete populations, but it is clear from these results that whole-genome sequence-based surveys tracking *Fibrobacter* populations through space and time are needed in order to fully understand the ecology and diversity of these fiber-degrading bacteria in their native environments.

The evolutionary relationships predicted from our concatenated alignment of 99 essential proteins are largely in agreement with what we, and others, have reported previously for the genus *Fibrobacter* (17, 29, 37), with our strains clearly resolving into two discrete lineages corresponding with previously described strains of F. succinogenes and F. intestinalis. Moreover, four major clades were readily identified in the phylogeny, including three (clades A to C) composed of F. succinogenes strains and one (clade D) containing all strains of F. intestinalis. Importantly, all of the phylotype designations previously identified using only full-length 16S rRNA gene sequences were preserved in our concatenated gene phylogeny produced here. However, the higher resolution obtained from our concatenated gene phylogeny allowed the detection of finer levels of phylogenetic diversity suggestive of ecologically discrete populations present within several of the previously reported *Fibrobacter* phylotypes (32). Placements of the *Fibrobacter* phylotypes with respect to each other were similar to what was predicted previously (17), except that strains from phylotype Fs IV are predicted to share a more recent common ancestor with strains of Fs I than was originally predicted from the 16S rRNA gene sequences alone.

In addition to being among the most actively cellulolytic mesophilic bacteria known, *Fibrobacter* has attracted interest due to its unusual strategy for PCWP degradation (18, 38, 39). Unlike many other anaerobic cellulose-degrading bacteria, which are found in the class *Clostridia*, stains of *Fibrobacter* do not produce a cellulosome to facilitate PCWP degradation (18). Homologs of genes for scaffoldins, cohesin modules, and essential cellulosomal components were similarly absent from the *Fibrobacter* genomes analyzed here. However, comparisons of CAZyme gene content data between our genomes suggested that the general mechanism of PCWP degradation is conserved among members of the genus *Fibrobacter*. Genes for the major families of cellulases (GH5, GH8, GH9, and GH45) and hemicellulases (GH10, GH26, and GH43), typical of previously described *Fibrobacter* genomes (18, 31), were present in all strains. While cellulases tended to occur without any identifiable associated CBMs, hemicellulase modules tended to co-occur with predicted CBM6s and CBM35s.

Unusual among the *Fibrobacter* cellulases was the identification of a single gene in all 40 *Fibrobacter* genomes predicted to encode a CBM11 along with a GH51 module. This fits the profile of the Cel51A protein, also known as EG2 and CelF, a major cellulose-binding endoglucanase found in the outer membrane of F. succinogenes S85 (35, 40). Cel51A accounts for a large fraction of the total endoglucanase activity produced by S85 (41, 42), and a global transcriptome analysis of S85 indicates that this gene is among the most highly expressed and constitutive (74). These results support the idea of a particularly important role for this protein in PCWP degradation by *Fibrobacter*.

A unique feature of the F. succinogenes cellulase system is the absence of processive exocellulases from the CAZyme GH6, GH7, and GH48 families (31, 39). Exocellulases within F. succinogenes exist as atypical members of the GH9 family (43). Similarly, no processive exocellulases were identified within the GH6, GH7, or GH48 CAZymes in the *Fibrobacter* genomes analyzed here. The distinctive mechanism of PCWP degradation by *Fibrobacter* likely also involves fibro-slime domain-containing proteins, which have not been identified in any other bacteria and are distantly related to proteins produced by the slime mold Dictyostelium discoideum (34). Fibro-slime domain-containing proteins have been shown to bind cellulose and were originally identified in cellulose adherence-defective mutants of F. succinogenes S85 (44). Genes predicted to encode fibro-slime domains were identified in all 40 *Fibrobacter* genomes analyzed here, although the number of genes predicted to contain these domains ranged from as low as 3 to as high as 10 among individual *Fibrobacter* strains. It is unclear what role these proteins play in PCWP degradation other than assisting in adherence, although an interesting hypothesis is that they may be involved in a form of gliding motility (18).

Despite sharing similar general mechanisms for PCWP degradation, substantial differences were observed in the total number of CAZyme-annotated gene families among phylogenetic groups. The most extreme example of this involved comparisons among representatives of the two *Fibrobacter* species. Strains from all three phylogenetic clades of F. succinogenes exhibited considerable enrichment in total CAZyme genes as well as in CAZyme classes involved in PCWP degradation compared to strains of F. intestinalis. This enrichment in F. succinogenes genomes extended to several individual CAZyme families, particularly GHs. We have previously shown that phylotypes of F. succinogenes tend to predominate in the gastrointestinal tracts of ruminants and large hindgut-fermenting mammals (17). We speculate that a higher rate of fiber degradation by the gut microbiota in these hosts may provide selective pressure to increase the capacity for enzyme production by these bacteria, explaining the general increases across CAZyme family genes observed in F. succinogenes relative to F. intestinalis. This selection would likely not be limited to *Fibrobacter*, and it has been reported that rumen strains of other polysaccharide-degrading organisms generally have higher proportions of CAZymes than closely related phylotypes from less-fibrolytic microbial communities (45 – 47). Although beneficial in highly fibrolytic environments such as the rumen, the increased cost associated with synthesizing these additional enzymes might be detrimental in environments where the rate of fiber degradation is lower. This may explain why F. intestinalis, with its more streamlined CAZyme profile, is more abundant in the guts of animals with less-fibrolytic microbiota, e.g., pigs, apes, and rats. While screening for qualitative differences in the levels of utilization of different carbohydrates among *Fibrobacter* strains has yielded little predictive information connecting the phylogeny to the ecology of these organisms, the availability of axenic cultures of the strains analyzed here, and their genomes, provides an opportunity to more rigorously test this hypothesis by screening for potential quantitative differences in their rates of fiber degradation.

Differences in CAZyme gene content were not limited to comparisons between F. succinogenes and F. intestinalis but extended to comparisons among the three major phylogenetic clades of F. succinogenes (clades A to C). A similar general increase in the total number of CAZymes in clades A and B relative to clade C was observed, although not to the degree found for F. succinogenes versus F. intestinalis. Many of the major CAZyme families thought to be important in PCWP degradation by *Fibrobacter*, including families CE6, GH8, GH9, GH10, GH43, and GH45, were found at higher abundance in strains from clades A and B than in strains from clade C. Given that the *Fibrobacter* phylotypes in clades A and B predominate in the rumen (17), the lower number of genes coding for cellulases and hemicellulases may contribute to the lower relative abundance of phylotypes of F. succinogenes clade C in the rumen.

Differences in CAZyme gene content among the different *Fibrobacter* clades could also be connected to differences in diet among herbivorous hosts. Due to their specialized anatomy for processing plant biomass, ruminants generally consume a diet that is chemically and structurally more complex than that of hindgut-fermenting herbivores (2). These differences could result in higher levels of selection for diverse enzymatic activities, even within a given CAZyme family. Although the specific functional differences within the CAZyme families identified here remain to be determined, some support for the idea of substrate preferences based on differences across CAZyme families was observed among the three major clades of F. succinogenes. Disparities in the total number of PLs in the genomes of strains from clades A, B, and C were less dramatic than they were for other CAZyme classes, and no difference between clades A and C in total levels of PL abundance was observed. Moreover, a single CBM77 was commonly predicted to occur in strains of clade C, as well as in several strains of F. intestinalis, but was not identified in any strains of clades A and B. PLs cleave uronic acid containing polysaccharides, which make up the backbone of pectin, and the only known activity of CBM77 is in binding pectin (48). *Fibrobacter* strains solubilize pectin from intact forages, but there are conflicting reports regarding their ability to utilize this substrate for growth (49 – 51). The disparities in CAZymes predicted to target pectin among these phylotypes suggest that the ability to depolymerize this substrate may be of greater importance for these bacteria in the hindgut than in the rumen. In the rumen, degradation of pectins occurs rapidly and involves diverse species of bacteria as well as anaerobic fungi (52 – 56). As a result, the pressure on rumen *Fibrobacter* phylotypes to degrade pectic polysaccharides in order to access cellulose fibers may be less intense than it is for *Fibrobacter* phylotypes adapted to the hindgut.

Although the phenotypic differences among the *Fibrobacter* isolates investigated here were few, genomic and functional evidence for some diversity of carbon and nitrogen metabolic capabilities between rumen and hindgut phylotypes was observed. Previous comparisons of *Fibrobacteres* genomes revealed variations in the presence of the gene coding for pyruvate kinase (31). We observed a similar result in our genome comparison. This gene was absent from all strains, except for those closely related to the F. intestinalis type strain and most strains in clade C. The explanation for this variation is unclear, although strains with this gene tended to originate from hindgut-fermenting herbivores. The gene encoding pyruvate formate lyase was also absent from strains in clade C. The lack of this gene could explain why formate production was not detected in an analysis of fermentation products produced by these strains (17). Variations in the ability to grow on d-lactose were also observed among these *Fibrobacter* strains, although no clear genetic explanation for this phenotype was identified. Growth on lactose by F. succinogenes S85 has previously been attributed to extracellular β-galactosidase activity (57, 58), and this is further supported by our finding that none of the 40 *Fibrobacter* genomes contained a lactose permease. Although all 40 *Fibrobacter* genomes contained genes predicted to code for β-galactosidase, we cannot rule out potential differences in expression that might explain the phenotypic diversity observed. It is worth noting that 5 of the 6 strains demonstrating growth on d-lactose were of rumen origin, as it has recently been hypothesized that the ability of some *Fibrobacter* strains to utilize lactose provides them with a selective advantage in the developing calf rumen (58).

Genomic comparisons among these *Fibrobacter* strains revealed the presence of a contiguous stretch of genes predicted to code for two catalytic subunits and accessory proteins for urease. Screening for growth on urea demonstrated that all but one of the strains possessing the urease genes were capable of growth using urea as a sole nitrogen source. *Fibrobacter* strains capable of utilizing urea were isolated primarily from hindgut-fermenting herbivores and found in clade C of our phylogeny, although 2 of the 16 rumen strains also exhibited this phenotype. Endogenous compounds such as urea make up a larger fraction of the nitrogen sources available to microorganisms in the hindgut than in the rumen because the rumen microbiota can more readily utilize dietary sources before they are absorbed by the host (59). This may explain why this trait was common in strains of F. succinogenes isolated from large hindgut-fermenting herbivores but was less so in strains of F. succinogenes isolated from the rumen. It remains to be determined how *Fibrobacter* strain UWT2 was able to grow readily in media with urea as the sole source of nitrogen when none of the urease genes were identified in the genome of this strain, and further investigation is warranted to determine if this strain possesses a unique mechanism for accessing nitrogen from urea.

In this study, we extend our previous work describing the isolation and ecological distribution of diverse *Fibrobacter* strains by sequencing and performing the most extensive comparative analysis of *Fibrobacter* genomes from pure cultures available to date. Clear differences in CAZyme gene content were observed among these strains, and these differences were strongly correlated with a whole-genome phylogeny based on amino acid sequences of essential genes. These results suggest that CAZymes are particularly active targets for genome evolution in *Fibrobacter*. Our analysis also identified variation in carbon and nitrogen metabolism among phylotypes that likely contributes to the ecology of these bacteria. The 38 novel *Fibrobacter* genomes generated here, as well as the availability of axenic cultures for these strains, provide valuable tools for testing specific hypotheses in future work aimed at unraveling the mechanism of PCWP degradation by *Fibrobacter* as well as their role in maintaining a healthy and efficient herbivore gut.

## MATERIALS AND METHODS

### *Fibrobacter* strains and culture conditions.

Isolation of the *Fibrobacter* strains analyzed in this study, along with their general characteristics such as major fermentation acids produced and the exact media formulation for cultivation, was described elsewhere (17). For this study, *Fibrobacter* strains were typically cultivated at 39°C in an anaerobic atmosphere consisting of 5% H_2_ plus 20% CO_2_, with the balance N_2_. The growth medium used was a slightly modified version of the medium originally described by Scott and Dehority (60), with Sigmacell 20 (Sigma-Aldrich, St. Louis, MO) (0.5% [wt/vol]) used as the primary carbon source. Culture on soluble sugars was performed at a final concentration of 20 mM, with the exception of maltose, which was included at a final concentration of 10 mM. Screening for growth with 5 mM urea as the sole nitrogen source was performed using the aforementioned media with microcrystalline cellulose as the carbon source and the following modifications: decreased l-cysteine–HCl (from 1 g liter^−1^ to 0.1 g liter^−1^), no NH_4_Cl, no tryptone, and no yeast extract. All strains were capable of growth with 10 mM NH_4_Cl as the sole nitrogen source in this version of the medium. Positive growth was defined as growth in the absence of a reduction in turbidity upon outgrowth after at least two sequential 1% (vol/vol) transfers.

### Genomic DNA extraction.

High-quality genomic DNA was obtained using the Department of Energy (DOE) Joint Genome Institute (JGI) method for bacterial genomic DNA isolation using cetyltrimethylammonium bromide (CTAB) (http://jgi.doe.gov/user-program-info/pmo-overview/protocols-sample-preparation-information/). Briefly, cells from 10 ml of broth culture were recovered via centrifugation and resuspended in 740 µl TE buffer (10 mM Tris-HCl, 1 mM EDTA, pH 8.0). SDS (10% [40 µl]) and proteinase K (10 mg ml^−1^ [8 µl]) were added followed by incubation at 56°C for 1 h. Next, 100 µl 5 M NaCl and 100 µl of a CTAB (275 mM)/NaCl (700 mM) solution were added followed by incubation at 65°C for 10 min. The DNA was then sequentially extracted with chloroform, phenol:chloroform:isoamyl alcohol, and chloroform and precipitated with isopropanol. The DNA was resuspended in TE buffer, and contaminating RNA was removed with RNase I (Epicentre, Madison, WI). Genomic DNA (gDNA) was quantified using a BR double-stranded DNA (dsDNA) assay kit and a Qubit Fluorometer (Invitrogen, Carlsbad, CA) and checked for RNA contamination by agarose gel electrophoresis.

### Genome sequencing, assembly, and annotation.

Whole-genome sequences were generated from either 300- or 550-bp-insertion standard Illumina shotgun libraries (Illumina, San Diego, CA) and sequenced using either an Illumina HiSeq or a MiSeq platform at the University of Wisconsin (UW)—Madison Biotechnology Center or the Department of Energy Joint Genome Institute (DOE JGI) (Walnut Creek, CA). Five of these isolates were also subjected to sequencing on a PacBio RS platform (Pacific BioSciences, Menlo Park, CA) from SMRTbell libraries at the DOE JGI. Raw Illumina reads were quality filtered per JGI standard operating practice (SOP) protocol 1061 using BBTools (http://bbtools.jgi.doe.gov) and assembled using SPAdes, v3.9.0 (parameters: ––phred–offset 33 ––cov–cutoff auto –m 40 ––careful –k 25,55,95 ––12) (61). Contigs that were <1,000 bp in length were discarded. Raw PacBio reads were assembled using HGAP, v2.3.0_p5 (protocol version = 2.3.0 method=RS HGAP Assembly.3,smrtpipe.py v1.87.139483) (62). Genome assemblies were assessed for quality and completeness using CheckM v1.0.11 (63). General genomic features were identified and annotated using Prokka, v1.12 (64). Kyoto Encyclopedia of Genes and Genomes (KEGG) pathway annotations were determined using the BlastKOALA online annotation tool (http://www.kegg.jp/blastkoala/) for K number assignment (65). Carbohydrate-active enzyme (CAZyme) annotation was performed using HMMER, v3.1 (66), and the dbCAN database, v6 (67). CAZyme annotations with an E value of >1 × 10^−18^ and coverage of <0.35 were discarded, as recommended for bacteria (http://csbl.bmb.uga.edu/dbCAN/).

### Comparative and phylogenetic analyses.

The genome-wide average nucleotide identity (ANI) of shared genes was calculated using the ani.rb script from the Enveomics Collection (33). A distance matrix containing the ANI distances for all pairwise comparisons between *Fibrobacter* strains was used to perform complete-linkage hierarchical clustering using the R function hclust. Cluster membership was determined using a cutoff of 0.05, corresponding to 95% ANI, typical of species-level differentiation for bacteria (32). A phylogenetic model of the evolutionary relationships among the strains was constructed from concatenated alignments of 99 essential proteins (see Table S5 in the supplemental material) identified in all 40 *Fibrobacter* genomes using the HMM.essential.rb script from the Enveomics Collection (33). Amino acid sequences for each of the 99 essential proteins were aligned using Clustal Omega v1.2.4 (68) prior to concatenation by strain. A maximum likelihood phylogeny was inferred from the concatenated alignment using RAxML v8.2.11 (69, 70). The PROTGAMMAGTR substitution model was used with rapid bootstrapping. The resulting phylogenetic tree was midpoint rooted and visualized using Dendroscope v3.5.9 (71).

10.1128/mSphere.00593-18.5 TABLE S5 List of essential proteins. Download Table S5, PDF file, 0.1 MB. Copyright © 2018 Neumann and Suen. 2018 Neumann and Suen This content is distributed under the terms of the Creative Commons Attribution 4.0 International license.

### Statistical analysis of CAZyme gene content.

CAZyme gene annotations for all 40 *Fibrobacter* strains were combined into a single data frame in R v3.4.3 (https://www.r-project.org). CAZyme gene content was normalized to control for differences in genome size among the strains by dividing the counts by the total number of genes identified in the corresponding genome to calculate the relative abundance for each CAZyme family. Normalized counts were calculated by multiplying the relative abundance by 2,553, the number of genes identified in the smallest *Fibrobacter* genome (UWS4), and rounding to the nearest integer. Normalized counts for total CAZyme genes and the CAZyme classes were assessed for normality and homoscedasticity by visually examining histograms and performing Levene’s test of equality of variances using the function levene.test from the “lawstat” package. The 40 *Fibrobacter* strains were grouped into 4 major clades (A to D) based on the results of the maximum likelihood phylogeny inferred from the essential protein sequences.

Statistical differences in total CAZyme genes, and for each CAZyme class present in the *Fibrobacter* genomes, among the 4 major clades were assessed by performing analysis of variance (ANOVA) using the normalized counts. The effect size (eta squared) indicating the proportion of the total variance in the dependent variable, normalized count, explained by the independent variable, *Fibrobacter* clade, was calculated using the R function etaSquared from the “lsr” package. Failure to reject the null hypothesis of no difference in normalized counts among the clades justified proceeding with pairwise *t* tests, which were performed using the R function pairwise.t.test with Bonferroni’s correction to adjust for multiple comparisons and the pool.sd parameter set to false. Effect sizes for pairwise tests were assessed by calculating Glass’s delta, the mean difference between group 1 and group 2 divided by the standard deviation of the whole sample, using the R function cohen.d (pooled = F) from the “effsize” package. Statistical differences in normalized counts for individual CAZyme families among the *Fibrobacter* clades were investigated using nonparametric tests by first testing for an overall difference using the Kruskal-Wallis rank sum test and then following up with pairwise Wilcoxon rank sum tests when appropriate. Statistical significance among the results of the 72 Kruskal-Wallis rank sum tests, one for each CAZyme family identified in all 40 *Fibrobacter* strains, was determined using a false-discovery rate (FDR) less than 0.05 calculated according to the method of Benjamini and Hochberg (72). *P* values resulting from the pairwise Wilcoxon rank sum tests were adjusted using Bonferroni’s correction. The FDR was used as the criterion for significance for all tests where the numbers of comparisons were greater than 20 in order to limit type II errors.

Hierarchical clustering of the 40 *Fibrobacter* strains according to CAZyme gene content was performed by calculating the Euclidean distances among the strains from the matrix of CAZyme family relative abundances in the respective genomes and by complete-linkage clustering using the R functions dist and hclust, respectively. A dendrogram of the results was constructed using functions from the R packages “ggdendro” and “ggplot2.” Nonmetric multidimensional scaling (NMDS) analysis of the strains based on CAZyme gene content was performed using CAZyme family relative abundances as input for the metaMDS function from the R package “vegan” (73). Vectors corresponding to the relative abundances of individual CAZyme families were fitted to the two dimensions of the NMDS using the “vegan” function envfit. CAZyme families present in all 40 *Fibrobacter* genomes were identified and used to construct a clustered heatmap of individual deviations in CAZyme family relative abundance from the mean for each strain and conserved CAZyme family using the function pheatmap from the R package “pheatmap.” Plots were generated using the R package “ggplot2.” All means are reported with their respective standard deviations.

### Accession number(s).

Genome assemblies have been submitted to the National Center for Biotechnology Information and deposited in the GenBank and RefSeq databases (https://www.ncbi.nlm.nih.gov/genome/genomes/50666). Individual GenBank accession numbers are listed in Table S2.
