# Supplementary material for: The Phylogenomic Diversity of Herbivore-Associated Fibrobacter spp. Is Correlated to Lignocellulose-Degrading Potential
Source: mSphere. 2018 Dec 12;3(6):e00593-18. doi: 10.1128/mSphere.00593-18 (PMC6291624; doi:10.1128/mSphere.00593-18)
Supplement: TABLE S1 [file sph006182728st1.pdf]

Table S1. Fibrobacter strains for comparative genomics

Strain	Phylotype	Host (ID)	Source	Sample site
UWB1	Fs II	Bovine (6844)	rumen	Dairy Cattle Center (WI, USA)
UWB2	Fs I	Bovine (4270)	rumen	USDA Prairie du Sac (WI, USA)
UWB3	Fs I	Bovine (4291)	rumen	USDA Prairie du Sac (WI, USA)
UWB4	Fs I	Bovine (4413)	rumen	USDA Prairie du Sac (WI, USA)
UWB5	Fs II	Bovine (4413)	rumen	USDA Prairie du Sac (WI, USA)
UWB6	Fs II	Bovine (5871)	rumen	Dairy Cattle Center (WI, USA)
UWB7	Fs I	Bovine	rumen	UW Veterinary Hospital (WI, USA)
UWB8	Fs II	Bovine (5971)	rumen	UW Dairy Cattle Center (WI, USA)
UWB10	Fs II	Bovine	rumen	UW Veterinary Hospital (WI, USA)
UWB11	Fs I	Bovine (4280)	rumen	USDA Prairie du Sac (WI, USA)
UWB12	Fs I	Bovine (4296)	rumen	USDA Prairie du Sac (WI, USA)
UWB13	Fs I	Bovine (4297)	rumen	USDA Prairie du Sac (WI, USA)
UWB15	Fs II	Bovine (6107)	rumen	Dairy Cattle Center (WI, USA)
UWB16	Fs I	Bovine (6551)	rumen	UW Dairy Cattle Center (WI, USA)
UWCM	Fs IV	Colobus	feces	Milwaukee County Zoo (WI, USA)
UWEL	Fs VI	Elephant	feces	Milwaukee County Zoo (WI, USA)
UWH1	Fs V	Equine	feces	UW Charmany Instructional Facility (WI, USA)
UWH3	Fs V	Equine	feces	UW Charmany Instructional Facility (WI, USA)
UWH4	Fs II	Equine	feces	Roll Farm (WI, USA)
UWH5	Fs V	Equine	feces	Pursely Stables (WI, USA)
UWH6	Fs V	Equine	feces	UW Charmany Instructional Facility (WI, USA)
UWH8	Fs V	Equine	feces	UW Charmany Instructional Facility (WI, USA)
UWH9	Fs V	Equine	feces	UW Veterinary Hospital (WI, USA)
UWOS	Fi I	Ostrich	feces	Henry Vilas Zoo (WI, USA)
UWOV1	Fs II	Ovine	rumen	UW Meat & Muscle lab (WI, USA)
UWP2	Fs VII	Capybara	feces	Henry Vilas Zoo (WI, USA)
UWR1	Fs VI	Rhino	feces	Henry Vilas Zoo (WI, USA)
UWR2	Fs IV	Rhino	feces	Henry Vilas Zoo (WI, USA)
UWR3	Fs IV	Rhino	feces	Milwaukee County Zoo (WI, USA)
UWR4	Fs VI	Rhino	feces	Henry Vilas Zoo (WI, USA)
UWRM	Fi III	Rhesus Macaque	feces	UW Primate Research Center (WI, USA)
UWS1	Fi I	Porcine	feces	UW Animal Sciences (WI, USA)
UWS2	ND	Porcine	cecum	UW Meat & Muscle lab (WI, USA)
UWS3	Fi II	Porcine	cecum	UW Meat & Muscle lab (WI, USA)
UWS4	Fi III	Porcine	cecum	UW Meat & Muscle lab (WI, USA)
UWT1	Fs V	Malayan Tapir	feces	Henry Vilas Zoo (WI, USA)
UWT2	Fs II	Malayan Tapir	feces	Henry Vilas Zoo (WI, USA)
UWT3	Fs IV	Baird's	feces	Milwaukee County Zoo (WI, USA)
S85*	Fs I	Bovine	rumen	NA
NR9*	Fi I	Rat	cecum	NA

*Type strain
