# Supplementary material for: The Phylogenomic Diversity of Herbivore-Associated Fibrobacter spp. Is Correlated to Lignocellulose-Degrading Potential
Source: mSphere. 2018 Dec 12;3(6):e00593-18. doi: 10.1128/mSphere.00593-18 (PMC6291624; doi:10.1128/mSphere.00593-18)
Supplement: TABLE S2 [file sph006182728st2.pdf]

Table S2. Genome assembly statistics

Strain	Method	GenBank accession	Completeness	Genome size (Mb)	GC	# scaffolds	# predicted genes	Proteins
UWB1	Illumina HiSeq(UWBC)	GCA_002210325.1	98.9	3.601604	50.2	46	2925	2904
UWB2	Illumina Hiseq(UWBC)	GCA_002210425.1	100	3.528394	48.8	15	2907	2898
UWB3	Illumina Hiseq(UWBC)	GCA_002210405.1	100	3.570823	48.4	20	2892	2874
UWB4	Illumina Hiseq(UWBC)	GCA_002210345.1	100	3.586285	48.2	18	2915	2901
UWB5	Illumina Hiseq(UWBC)	GCA_002210295.1	100	3.361559	50.9	17	2717	2709
UWB6	Illumina MiSeq(JGI)	GCA_003148985.1	100	3.46817	50.2	26	2823	2806
UWB7	Illumina HiSeq(JGI)	GCA_900142945.1	100	3.812257	47.9	12	3110	3098
UWB8	Illumina HiSeq(JGI)	GCA_900129525.1	100	3.46935	50.2	30	2827	2810
UWB10	Illumina MiSeq(JGI)	TBD	100	3.479813	49.4	10	2785	2773
UWB11	Illumina HiSeq(JGI)	GCA_900143015.1	100	3.763481	46.8	9	3063	3055
UWB12	Illumina HiSeq(JGI)	GCA_900142485.1	100	3.826342	46.8	21	3096	3078
UWB13	Illumina MiSeq(JGI)	GCA_900177805.1	100	3.804669	47.9	10	3103	3096
UWB15	Illumina MiSeq(JGI)	GCA_900177705.1	100	3.464091	50.2	24	2821	2804
UWB16	Illumina HiSeq(JGI)	GCA_900215325.1	100	3.555739	48.5	9	2884	2874
UWCM	Illumina HiSeq(JGI)	GCA_900129735.1	99.94	3.784167	53.4	65	3122	3095
UWEL	Illumina HiSeq(JGI)	GCA_900142535.1	100	3.530042	49.1	62	2938	2910
UWH1	Illumina Hiseq(UWBC)	GCA_002210365.1	100	3.830823	48.9	96	3249	3234
UWH3	Illumina Hiseq(UWBC)	GCA_002210355.1	100	3.868941	48.8	62	3224	3197
UWH4	Illumina HiSeq(JGI)	GCA_900142475.1	98.9	3.696015	50.8	22	3083	3056
UWH5	Illumina HiSeq(JGI)	GCA_900142505.1	100	3.736655	49.1	59	3116	3081
UWH6	Illumina HiSeq(JGI)	GCA_900142465.1	100	3.921019	48.8	50	3273	3236
UWH8	Illumina HiSeq(JGI)	GCA_003268805.1	100	3.851655	49	97	3285	3246
UWH9	Illumina HiSeq(JGI)	GCA_900129715.1	100	3.784473	49	52	3174	3140
UWOS	Illumina HiSeq(JGI)	GCA_900142455.1	99.94	3.242118	48	93	2879	2829
UWOV1	Illumina HiSeq(JGI)	GCA_900142495.1	98.9	3.687051	50.3	29	3062	3054
UWP2	Illumina HiSeq(JGI)	GCA_900141705.1	100	3.234975	53.4	72	2719	2690
UWR1	Illumina MiSeq(JGI)	GCA_003253655.1	100	3.606749	48.7	100	3037	2988
UWR2	Illumina Hiseq(UWBC)	GCA_002210285.1	100	3.278051	53.4	20	2721	2711
UWR3	Illumina HiSeq(JGI)	GCA_900143055.1	99.94	3.546738	53.8	20	2929	2918
UWR4	Illumina MiSeq(JGI)	GCA_003149045.1	100	3.60147	48.7	93	3004	2981
UWRM	Illumina HiSeq(JGI)	GCA_003003115.1	99.94	2.85944	44.8	69	2578	2521
UWS1	PacBio(JGI)	GCA_002300445.1	99.94	3.380004	48.1	2	2950	2934
UWS2	Illumina MiSeq(JGI)	GCA_003014635.1	100	2.947569	48.9	77	2661	2635
UWS3	PacBio(JGI)	GCA_002797675.1	100	3.127022	49.2	1	2836	2828
UWS4	Illumina MiSeq(JGI)	GCA_003148885.1	98.84	2.849881	44.8	78	2553	2502
UWT1	PacBio(JGI)	GCA_002846255.1	100	4.019388	49.3	1	3432	3422
UWT2	Illumina HiSeq(JGI)	GCA_900142545.1	100	3.493574	50.2	49	2785	2785
UWT3	PacBio(JGI)	GCA_900230125.1	99.94	3.810443	53.8	5	3225	3217
S85*		GCA_000024665.1	100	3.842635	48.1	1	3126	3119
NR9*	PacBio(JGI)	GCA_002300465.1	99.39	3.390891	47.9	1	2992	2979

*Type strain

JGI = DOE Joint Genome Institute

UWBC = University of Wisconsin Biotech Center

TBD = to be determined
