# Supplementary material for: The Phylogenomic Diversity of Herbivore-Associated Fibrobacter spp. Is Correlated to Lignocellulose-Degrading Potential
Source: mSphere. 2018 Dec 12;3(6):e00593-18. doi: 10.1128/mSphere.00593-18 (PMC6291624; doi:10.1128/mSphere.00593-18)
Supplement: TABLE S3 [file sph006182728st3.pdf]

Table S3. CAZyme families summary stats

CAZy Family	total count	mean (normalized)	strains positive	Q1 percentile (normalized)	median (normalized)	Q3 percentile (normalized)
GT2	725	15.7	40	13	15	18
CBM6	648	14	40	12	15	17
GH5	523	11.3	40	10	11.5	12
GT4	452	9.925	40	8	10	12
GH43	403	8.65	40	8	9	10.25
CBM35	378	8.075	40	7	8	11
GH9	341	7.325	40	6	7	9
PL1	264	5.75	40	5	6	6
GH8	215	4.725	40	4	5	6
GH10	201	4.3	40	3	4	6
GH26	172	3.675	40	3	4	5
CBM4	153	3.3	40	3	3	4
CE6	149	3.25	40	2	3	4
GH13	147	3.2	40	3	3	4
GH45	146	3.125	40	2	3	4
GH16	141	3	40	3	3	4
GH30	134	2.9	40	2	3	4
GH57	117	2.575	40	2	3	3
CBM51	113	2.5	40	2	2.5	3
GH23	112	2.475	40	2	2	3
GT51	108	2.45	40	2	2	3
CE1	106	2.3	37	2	3	3
CE12	104	2.475	40	2	2	3
PL11	93	2.25	40	2	2	3
GH11	91	2.125	40	1	2	3
GH2	73	1.8	40	2	2	2
GT32	70	1.65	37	1	2	2
CE10	68	1.525	40	1	1.5	2
GH3	68	1.6	33	1	2	2
GH18	67	1.675	40	1	2	2
GT5	67	1.65	40	1	2	2
PL9	58	1.35	30	0.75	1	2
CE15	50	1.225	32	1	1	2
GH53	48	1.2	35	1	1	2
CE2	45	1.125	40	1	1	1
GH116	41	1.025	40	1	1	1
CBM11	40	1	40	1	1	1
CE11	40	1	40	1	1	1
GH51	40	1	40	1	1	1
GH74	40	1	40	1	1	1
GH77	40	1	40	1	1	1
GH94	40	1	40	1	1	1
GT19	40	1	40	1	1	1
GT28	40	1	40	1	1	1
GT35	40	1	40	1	1	1
CBM48	39	0.975	39	1	1	1
GH44	36	0.9	36	1	1	1
GH27	35	0.875	35	1	1	1
GT30	34	0.85	34	1	1	1
CE8	33	0.825	33	1	1	1
GH127	33	0.825	33	1	1	1
GH141	33	0.825	33	1	1	1
GH95	33	0.825	33	1	1	1
PL10	33	0.825	33	1	1	1
PL14	32	0.8	30	0.75	1	1
GT26	23	0.575	22	0	1	1
GH39	22	0.55	22	0	1	1
GT21	21	0.525	21	0	1	1
GT94	21	0.525	19	0	0	1
CBM77	18	0.45	18	0	0	1
GT8	13	0.275	8	0	0	0
CE4	12	0.3	12	0	0	1
GH54	12	0.3	12	0	0	1
PL12	12	0.3	12	0	0	1
GT1	10	0.25	10	0	0	0.25
CBM61	9	0.225	9	0	0	0
PL8	9	0.225	9	0	0	0
GT14	7	0.175	7	0	0	0
GT56	6	0.15	6	0	0	0
GT27	1	0.025	1	0	0	0
GT46	1	0.025	1	0	0	0
GT92	1	0.025	1	0	0	0
