# Supplementary material for: The Phylogenomic Diversity of Herbivore-Associated Fibrobacter spp. Is Correlated to Lignocellulose-Degrading Potential
Source: mSphere. 2018 Dec 12;3(6):e00593-18. doi: 10.1128/mSphere.00593-18 (PMC6291624; doi:10.1128/mSphere.00593-18)
Supplement: TABLE S4 [file sph006182728st4.pdf]

Table S4. Statistical differences in CAZyme Classes

**Total CAZymes**				
ANOVA	p-value	eta squared		
	8.16E-14	0.827522		
pairwise t-tests	p-value	Glass's delta#	Group 1 mean	Group 2 mean
A vs B	0.03764	1.261902	182.1429	200
A vs C	0.02978	1.249686	182.1429	165.7
A vs D	1.10E-09	5.173736	182.1429	124.5714
B vs C	0.00029	2.60686	200	165.7
B vs D	3.30E-08	5.330275	200	124.5714
C vs D	5.30E-06	3.125843	165.7	124.5714
**CAZymes by Class**				
**CBM**				
ANOVA	p-value	eta squared		
	3.35E-12	0.7877001		
pairwise t-tests	p-value	Glass's delta#	Group 1 mean	Group 2 mean
A vs B	1	0.6671121	33.42857	35.11111
A vs C	0.344	0.8070824	33.42857	30.5
A vs D	8.70E-09	4.449364	33.42857	18.85714
B vs C	0.031	1.270772	35.11111	30.5
B vs D	1.60E-08	6.444555	35.11111	18.85714
C vs D	4.50E-06	3.208645	30.5	18.85714
**CE**				
ANOVA	p-value	eta squared		
	< 2.2E-16	0.9119973		
pairwise t-tests	p-value	Glass's delta#	Group 1 mean	Group 2 mean
A vs B	0.00041	2.236341	15	18.55556
A vs C	0.00734	1.488417	15	13.4
A vs D	1.80E-06	8.958521	15	7.142857
B vs C	6.70E-06	4.79601	18.55556	13.4
B vs D	2.20E-09	7.178255	18.55556	7.142857
C vs D	3.30E-06	5.820773	13.4	7.142857
**GH**				
ANOVA	p-value	eta squared		
	6.04E-15	0.8508508		
pairwise t-tests	p-value	Glass's delta#	Group 1 mean	Group 2 mean
A vs B	0.0981	1.036675	83.85714	93.44444
A vs C	0.0147	1.431837	83.85714	75.7
A vs D	9.50E-11	6.158124	83.85714	49.28571
B vs C	0.0015	3.114712	93.44444	75.7
B vs D	2.30E-07	4.774886	93.44444	49.28571
C vs D	6.30E-08	4.636544	75.7	49.28571
**GT**				
ANOVA	p-value	eta squared		
	1.58E-03	0.3426113		
pairwise t-tests	p-value	Glass's delta#	Group 1 mean	Group 2 mean
A vs B	1	0.2983944	38.21429	38.88889
A vs C	0.0813	1.340286	38.21429	33.7
A vs D	0.5949	0.6306452	38.21429	41.28571
B vs C	0.0066	1.540575	38.88889	33.7
B vs D	0.7085	1.060178	38.88889	41.28571
C vs D	2.00E-03	2.252189	33.7	41.28571
**PL**				
ANOVA	p-value	eta squared		
	5.46E-08	0.6340879		
pairwise t-tests	p-value	Glass's delta#	Group 1 mean	Group 2 mean
A vs B	0.023	1.666752	11.64286	14
A vs C	1	0.7043401	11.64286	12.4
A vs D	9.80E-05	1.767617	11.64286	8
B vs C	0.089	1.488417	14	12.4
B vs D	4.80E-07	4.242641	14	8
C vs D	5.80E-07	4.093146	12.4	8

absolute value
