# Supplementary material for: The Phylogenomic Diversity of Herbivore-Associated Fibrobacter spp. Is Correlated to Lignocellulose-Degrading Potential
Source: mSphere. 2018 Dec 12;3(6):e00593-18. doi: 10.1128/mSphere.00593-18 (PMC6291624; doi:10.1128/mSphere.00593-18)
Supplement: TABLE S5 [file sph006182728st5.pdf]

Table S5. Essential protein list

Protein	Annotation
GrpE	GrpE
Methyltransf_5	MraW methylase family
PGK	Phosphoglycerate kinase
Ribosomal_L10	Ribosomal protein L10
Ribosomal_L23	Ribosomal protein L23
Ribosomal_L3	Ribosomal protein L3
Ribosomal_L4	Ribosomal protein L4/L1 family
Ribosomal_L5	Ribosomal protein L5
Ribosomal_L6	Ribosomal protein L6
Ribosomal_S11	Ribosomal protein S11
Ribosomal_S13	Ribosomal protein S13/S18
Ribosomal_S17	Ribosomal protein S17
Ribosomal_S8	Ribosomal protein S8
TIGR00001	rpmI_bact: ribosomal protein L35
TIGR00002	S16: ribosomal protein S16
TIGR00009	L28: ribosomal protein L28
TIGR00012	L29: ribosomal protein L29
TIGR00019	prfA: peptide chain release factor 1
TIGR00043	TIGR00043: metalloprotein, YbeY/UPF0054 family
TIGR00059	L17: ribosomal protein L17
TIGR00061	L21: ribosomal protein L21
TIGR00062	L27: ribosomal protein L27
TIGR00064	ftsY: signal recognition particle-docking protein FtsY
TIGR00082	rbfA: ribosome-binding factor A
TIGR00086	smpB: SsrA-binding protein
TIGR00092	TIGR00092: GTP-binding protein YchF
TIGR00115	tig: trigger factor
TIGR00116	tsf: translation elongation factor Ts
TIGR00152	TIGR00152: dephospho-CoA kinase
TIGR00158	L9: ribosomal protein L9
TIGR00165	S18: ribosomal protein S18
TIGR00166	S6: ribosomal protein S6
TIGR00168	infC: translation initiation factor IF-3
TIGR00234	tyrS: tyrosine--tRNA ligase
TIGR00337	PyrG: CTP synthase
TIGR00344	alaS: alanine--tRNA ligase
TIGR00362	DnaA: chromosomal replication initiator protein DnaA
TIGR00389	glyS_dimeric: glycine--tRNA ligase
TIGR00392	ileS: isoleucine--tRNA ligase
TIGR00409	proS_fam_II: proline--tRNA ligase
TIGR00414	serS: serine--tRNA ligase
TIGR00420	trmU: tRNA (5-methylaminomethyl-2-thiouridylate)-methyltransferase
TIGR00422	valS: valine--tRNA ligase
TIGR00435	cysS: cysteine--tRNA ligase
TIGR00436	era: GTP-binding protein Era
TIGR00442	hisS: histidine--tRNA ligase
TIGR00459	aspS_bact: aspartate--tRNA ligase
TIGR00460	fmt: methionyl-tRNA formyltransferase
TIGR00468	pheS: phenylalanine--tRNA ligase, alpha subunit
TIGR00472	pheT_bact: phenylalanine--tRNA ligase, beta subunit
TIGR00487	IF-2: translation initiation factor IF-2
TIGR00496	frr: ribosome recycling factor
TIGR00575	dnlj: DNA ligase, NAD-dependent
TIGR00631	uvrb: excinuclease ABC subunit B
TIGR00663	dnan: DNA polymerase III, beta subunit
TIGR00810	secG: preprotein translocase, SecG subunit
TIGR00855	L12: ribosomal protein L7/L12
TIGR00922	nusG: transcription termination/antitermination factor NusG
TIGR00952	S15_bact: ribosomal protein S15
TIGR00959	ffh: signal recognition particle protein
TIGR00963	secA: preprotein translocase, SecA subunit
TIGR00964	secE_bact: preprotein translocase, SecE subunit
TIGR00967	3a0501s007: preprotein translocase, SecY subunit
TIGR00981	rpsL_bact: ribosomal protein S12
TIGR01009	rpsC_bact: ribosomal protein S3
TIGR01011	rpsB_bact: ribosomal protein S2
TIGR01017	rpsD_bact: ribosomal protein S4
TIGR01021	rpsE_bact: ribosomal protein S5
TIGR01024	rplS_bact: ribosomal protein L19
TIGR01029	rpsG_bact: ribosomal protein S7
TIGR01031	rpmF_bact: ribosomal protein L32
TIGR01032	rplT_bact: ribosomal protein L20
TIGR01044	rplV_bact: ribosomal protein L22
TIGR01049	rpsJ_bact: ribosomal protein S10
TIGR01050	rpsS_bact: ribosomal protein S19
TIGR01059	gyrB: DNA gyrase, B subunit
TIGR01063	gyrA: DNA gyrase, A subunit
TIGR01066	rplM_bact: ribosomal protein L13
TIGR01067	rplN_bact: ribosomal protein L14
TIGR01071	rplO_bact: ribosomal protein L15
TIGR01079	rplX_bact: ribosomal protein L24
TIGR01164	rplP_bact: ribosomal protein L16
TIGR01169	rplA_bact: ribosomal protein L1
TIGR01171	rplB_bact: ribosomal protein L2
TIGR01391	dnaG: DNA primase
TIGR01393	lepA: GTP-binding protein LepA
TIGR01632	L11_bact: ribosomal protein L11
TIGR01953	NusA: transcription termination factor NusA
TIGR02012	tigrfam_recA: protein RecA
TIGR02013	rpoB: DNA-directed RNA polymerase, beta subunit
TIGR02027	rpoA: DNA-directed RNA polymerase, alpha subunit
TIGR02191	RNaseIII: ribonuclease III
TIGR02350	prok_dnaK: chaperone protein DnaK
TIGR02386	rpoC_TIGR: DNA-directed RNA polymerase, beta' subunit
TIGR02397	dnaX_nterm: DNA polymerase III, subunit gamma and tau
TIGR02432	lysidine_TilS_N: tRNA(Ile)-lysidine synthetase
TIGR02729	Obg_CgtA: Obg family GTPase CgtA
TIGR03263	guanyl_kin: guanylate kinase
tRNA-synt_1d	tRNA synthetases class I (R)
